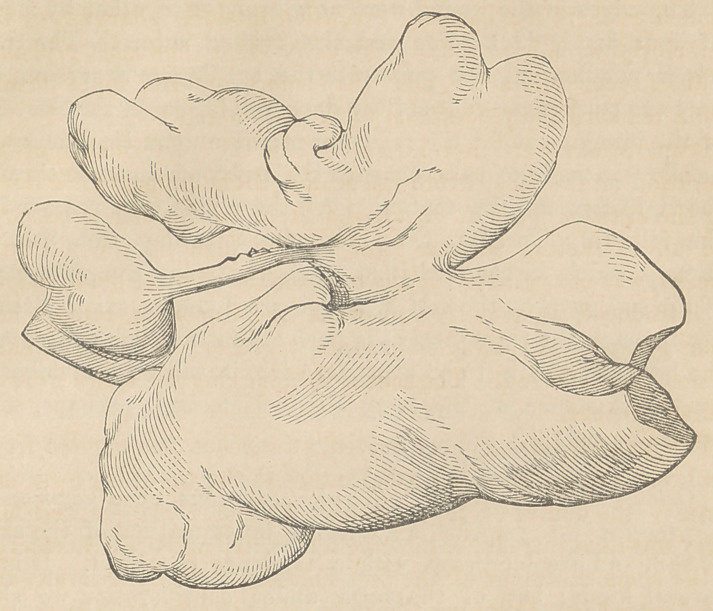# Pennsylvania College, Ninth below Locust Street

**Published:** 1852-11

**Authors:** W. H. Gobrecht


### CLINICAL REPORTS.

*Pennsylvania College, Ninth below Locust street. Service of* Professor Gilbert.

Reported by W. H. Gobrecht, M. D.

*Oct. 2d,* 1852.—Case I. *Contraction of long flexor Tendon of Thumb, and operation.*—The case now brought before the Class, is one which had been contraction of the flexor tendons of all the fingers and thumb of the right hand. The patient, Lydia T——, aged 16, a healthy looking young girl, states that about three and a half years since the contractile affection was first perceived, without any previous injury.

About twenty months ago, one or two black spots were noticed just behind the knuckles of that hand, which were attended with burning pain, and in two or three days the skin, thus marked, sloughed out. Then other similar spots followed in the course of the arm, and in the position of all such spots cicatrices are now to be found.

This contraction of the tendons is unlike either torticollis or varus, in which the deformity depends upon rigid atrophy of the muscle; but on the contrary it is spastic, and arises from an abnormal condition of the upper portion of the spinal cord, probably from chronic inflammation of the investing membrane, and therefrom results a derangement of the nervous supply. In support of this view, we find that the patient has extreme tenderness of the spine in the interscapular and cervical regions. If this irritation could be obviated, then the contraction would be relieved; but attempts at this relief have been made without any evident effect. The tendons of the fingers were cut a year since by an eminent surgeon of this city, followed by great relief. The thumb was also operated upon by cutting most probably the anterior tendon of the flexor brevis pollicis, with additional relief, and a digitated iron splint had been employed to retain the fingers and thumb extended. The thumb has since then resumed its old position and its deformity has increased, so that whilst the fingers are flexile and extensile, the point of the thumb has been gradually and so firmly buried in the palm of the hand that ulceration has been set up attended with great and increasing pain, and notwithstanding a well stuffed pillow has been placed beneath the offending organ to obviate the pressure, its benefit is so small that the patient had some four weeks since a violent spasm of long duration, evidently the result of said pressure. An operation is now demanded for the patient's relief. Firstly, on account of the spinal irritation; secondly, because the hand is rendered totally useless from the position of the thumb across its palm, so that amputation of the thumb has been advised by a practitioner who has previously examined the case. But the intention of Prof. Gilbert is to save to the patient, if possible, this most valuable portion of the prehensile member, and therefore, it is proposed to continue the general treatment addressed to the nervous centres, or establish new as may be required, and to obviate the local difficulty, by the division of the long flexor tendon of the thumb above the wrist.

The operation was then performed as follows: The patient being seated, a small incision was made on the ulnar side of, and parallel with the tendon of the flexor carpi radialis, just above the anterior annular ligament of the wrist. A dull tenotomy knife was then introduced, and the point passed beneath the tendon of the flex. carp. radialis and radial vessels, being then turned edge downwards pressure was made against the bone and towards the radial side of the fore arm.

The dulness of the knife obviated any injury which might accrue to the median nerve by a sharply cutting edge from which it could not recede as from a blunted one, should they chance to come in contact.

On the complete division of the tendon the thumb was instantly relaxed. The wound was then closed by an adhesive strip, and the thumb and fingers fully extended by means of the iron splint before used.

On *Oct. 6th* the patient was again presented to the class. She could extend the thumb perfectly, and flex it slightly voluntarily, probably by means of the flexor ossis metacarpi pol, which still remains, and perhaps the flexor brevis pol.

The constitutional means now prescribed were—

℞. Extr. Conii ℈j, ft. pil. No. xij. S. One thrice daily and two at bed time. Also the following anti-spasmodic tonic.

℞. Pulv. Cinchonæ Rubr. ℥j.

" Rad. Valerianæ ℥j.

" Sem. Cardamomi ʒj.

M. ft. chart No. IV. S. To each powder add half a pint of boiling water. Dose, two tablespoonfuls thrice daily.

To these we add the local application of creasote by means of a brush to the side of the tender portions of the spinal column, as a counter-irritant. By these means we may hope to obviate much of the existing trouble.

The patient left town in ten days after the operation greatly improved in condition; the thumb and fingers extended upon the splint.

Case II. *Enlarged Tonsils.*—These are apt to follow *repeated* attacks of quinsy or cynanche tonsillaris. If the inflammation of the tonsil is acute, pus generally forms and is discharged, the gland returning to its original size; but after frequent attacks, the subsequent chronic inflammation produces enlargement and hardening, obstructing deglutition, impeding respiration, and changing the voice. In such cases, before an operation is determined upon, we should wait until the natural color and general appearance of the fauces have been restored.

A variety of instruments has been employed for the removal of enlarged tonsils. (Here the combined scissors and forceps and every variety of the guillotine plan of instrument were exhibited.) The case presented was one in which a plan of treatment differing from that ordinarily employed in this country had been recommended to the patient by her former attendant. It was proposed to remove the tonsil by the wire *ligature* applied by means of the canula. In this method of operating the instrument be worn three or four days, and the patient subjected to the tedious and unpleasant process of sloughing. The fear of hæmorrhage appears to have originated this treatment, but this is not to be much regarded if the tonsil be excised on a line with the lateral palatine half arches, without drawing it into the fauces. This patient was instantly relieved of the major part of the right tonsil by means of Fahnestock's guillotine instrument with but very inconsiderable venous hæmorrhage.

Case III.—In presenting to the class an interesting case of *Polypus Nasi* with the operation, Prof. Gilbert made the following prefatory remarks:

Nasal polypi are tumors of one or both nasal cavities, which if allowed to remain, will increase and block up the affected nostril, and displace the septum narium, thus obstructing the other nostril, while the continued growth may produce deformity of the cheek, prevent the passage of the tears by pressing on the nasal duct, and perhaps cause even death by pressure on the brain.

Polypi may be divided into 1st, *The Soft*, which includes 1. The *Mucous* polypus, of which kind Dr. G. removed twenty four of small size from a patient at one sitting. 2. *Encysted*, which is a modification of the mucous.

2d. The *Hard*, which includes—1. The *Fibrous*, the base of which is broader and firmer than the mucous polypus, and arises probably from the periosteum. 2. *Fleshy*, or sarcomatous, containing a larger number of vessels. 3. *Malignant* or *Medullary*, which may be so per se, or by a degeneration of the fibrous variety.

The case of polypus before the class is supposed to be of the *Fibrous* description. The patient, J. P——, an Irishman, aged 36, a laborer, states that about six years since his nose became a ffected, and that ever since that timehe has experienced gradually increasing difficulty in breathing, up to about a year and a half back, since which time he has had no use of either nostril in the performance of respiration, except perhaps a very little on rising in the morning. Constant head-ache troubles him and stillicidium lachrymarum is present. The nasal bones and nasal processes of the superior maxillaries are expanded, and the latter encroach on the orbit in such a manner as to evert both eyes, giving to the patient the appearance presented by a case of double divergent strabismus, as is shown in the accompanying cut from a daguerreotype by Laughlin.

It will here be seen that the lower part of the nasal bones form the upper part of the arch of the large tumor, which thus occupies the centre of the face, terminated above by the comparatively narrow bridge and below by the contracted apex of the nose.

The nostrils on examination are found filled with a resisting, soft, organized substance. Evidently large polypi.

In diagnosticating the variety of polypus with which we have to deal, we must act somewhat upon the method by exclusion, thus: The disease cannot be of a malignant nature, inasmuch as there is no cachexia. It has lasted for six years, with no inconvenience except its increase. There is no offensive discharge, and the polypi do not bleed. The affection must therefore be *benign*. The polypi are probably not of the mucous or encysted kind, inasmuch as there has been no extension into the pharynx, or far anteriorly, as is here usually the case. It is thus most probably of the fibrous character, and the tumors are no doubt firmly connected with the periosteum of the turbinated bones.

Preparations were therefore made for the performance of an operation on the most extensive scale. Bone nippers for enlarging the cavities were at hand, should it be deemed necessary, and an extractor, with a joint on the plan of the obstetric forceps, was constructed for the occasion, so that the blades might be introduced separately, and when the tumor or tumors had been grasped, the handles brought together and the ordinary torsion then effected. The means for plugging the nares were of course present.

The patient having been seated, an incision was carried from the lower extremity of the nasal suture to the tip of the nose and down to the centre of the septum. A long, sharp-pointed bistoury was then introduced into the left nostril and made to emerge at the commencement of the first incision, then by a downward and forward cut, following the track of that incision, the nostril of the same side was laid open. The right was treated in a corresponding manner. Large polypous masses were thus exposed, and the prepared forceps were introduced into the right cavity. After the breaking up of a few smaller masses and several ineffectual attempts at the larger one, a double lobulated tumor with a central plate of bone was with some difficulty withdrawn.

This mass is figured of life size. [*See next page.*]

It presented very much the shape and size of two large oysters connected firmly together, containing various large and small sacs which discharged a greater or smaller amount of flaky, whitish pus.

One or two smaller polypi were now extracted and, several small plates of bone came away attached to their bases.

On now examining the left nasal cavity an encysted polypus was broken up, out of which gushed about two drachms of pus, and then one or two smaller polypi were extracted; on examination with the finger it was found that the lower part of the vomer had been pushed so far into this cavity that it was almost completely occluded, so that but few polypi here existed, this nostril being in great part only secondarily obstructed.

The right posterior naris was then plugged by the following simple and ingenious method:—ordinary bonnet wire of about 30 inches in length (such as is used by milliners to stiffen the bows of bonnets, and which can therefore be obtained at a moment's notice, when the more beautiful but less easily transportable canula of Belloc is not at hand) was doubled upon itself, passed into the anterior nares, and on appearing posteriorly it was seized by the forceps and drawn forward; the plug of sponge was then introduced within the loop of the double extremity and the wire twisted upon itself in such a manner as to firmly secure the tampon, which was then,—first having attached a cord to it by which it might, if necessary, be withdrawn by the mouth,—brought up against and into the posterior naris which it firmly closed; another sponge, in ordinary cases, is then placed against the anterior naris, and the free ends of the wire knotted strongly upon it, when the cavity is effectually deprived of any external communication; but here, in consequence of the anterior incisions, no such pressure could be employed, and consequently adhesive straps had to be relied on. The left posterior naris did not require to be plugged.

The edges of the wound were now brought together by means of four fine gold needles and the twisted suture. The three upper needles did not penetrate the cartilaginous septum, but only the contiguous edges of the flaps, relying upon the elasticity of the integument for the requisite pressure upon it. The lowest needle was made to pass beneath the point of the nose through the right flap and the septum. All these pins were removed on the following Tuesday, Oct. 5th. On Saturday, Oct. 9th, the threads which surrounded them came away, and complete union of the soft parts by the first intention was found to exist. Breathing through the right nostril is perfect, but not quite so free in the left, and this is to be accounted for, it will be remembered, by the existence of the bony obstruction, and, perhaps, some concealed polypi.

*October 6th.*—CASE IV. *Cellulo-Cutaneous,* or *Phlegmonous Erysipelas.*—Erysipelas is a diffused inflammation of the skin, or skin and areolar tissue, with a tendency to spread. It is of several kinds; but of the form under consideration we shall merely state, that it extends from the integument down to the subjacent areolar tissue, and thence communicates itself to the muscular and tendonous investments. Abscess may then form and extend itself; depression of the vital powers ensues; sloughing of the tissues with a discharge of ichor and thin sanious pus, and frequently death may result.

In the case presented, an adult male, who appeared for treatment upon Wednesday last, and was seen by many of you at that time, the back of the right hand was immensely swollen, tense and red, as were also the parts about the metacarpo-phalangeal articulation of the thumb. This redness and swelling had extended up the arm, and seemed disposed to spread. *Creasote* (pure) was applied over the entire inflamed surface, by means of the camel's hair pencil, and a poultice ordered, inasmuch as an abscess seemed almost inevitable. Upon the third day—namely, October 2d, he re-appeared, and the irritation and swelling, by the counter-irritant action of the creasote, had greatly diminished. The creasote was then re-applied, and the poultice continued. To-day, Oct. 6th, we find nearly all the pain and redness gone; the swelling greatly diminished, as shown by the wrinkling of the skin, and all fears of an abscess have vanished. We now re-apply the creasote which has at no time given him very great pain, expecting to find him, at his next visit, perfectly relieved, having only, perhaps, some remaining stiffness of the joints.

The case in point demonstrates most satisfactorily the value of pure creasote, as a local application, in the treatment of erysipelas.

CASE V. *Exomphalos, or Umbilical Hernia.*—This may be either *congenital* or *non-congenital.* In the former, the intestine or omentum is protruded into the umbilical cord; it is to be recognized by the abnormal enlargement of this structure, the extruded substance forming a conical tumor within it, near to the umbilicus, and for this occurrence we must be constantly on our guard. In the non-congenital, occurring generally in the infant, as in the present case, the tumor forms gradually from some such cause as has here operated. These herniæ may be either reducible or irreducible, but are seldom strangulated.

Francis M'C——, aged 11 months, was now presented, with the following history drawn from the statement of the mother. A tumor appeared at the umbilicus at about six months after birth, which seems to have arisen from muscular exertion, since the child was in the habit of implanting his heels firmly against any resisting body, as the knees of his nurse, and throwing his head and shoulders strongly back, and thus, by extending the spine, projecting the abdomen; the resistance of its muscular walls then acting forcibly on the contained viscera, a part were extruded gradually at the weakest dilatable point. By pressing upon the tumor it is easily reduced, and must be retained by means of a small semi-globular mass of wood, wrapped with narrow bandage, to increase its bulk and render it soft and equable in its pressure; this is placed over the weakened point in the abdomen, and retained by a circular bandage thrown about the body of the infant. This pad must be worn for some nine or twelve months, until the growth of the surrounding tissues has obliterated the abnormal opening.

CASE VI. George W——, aged 12, had both tonsils removed by means of Fahnestock's instrument.

CASE VII. *Cataract.*—Prof. Gilbert remarked that cataract may be either idiopathic or traumatic, and then proceeded to describe its varieties, together with the immediate causes of the disease and its effects. The different methods of operation for its relief were then detailed, illustrated by a variety of large diagrams. In the case presented, which was of the *soft* variety, of the left eye, occurring in an adult German, male, aged about 40, Prof. Gilbert preferred the form of operation known as Keratonyxis, in which the needle is introduced through some part of the cornea, and made to reach the obstructing lens through the anterior chamber of the eye. This method is perhaps best employed in first operations, where absorption is desired. We may thus effectually lacerate the capsule, the whole of the needle being visible during the entire operation, and all danger of wounding the ciliary body avoided.

The pupil was previously well dilated by means of a few drops of a solution of Atropia, gr. i. to f.ʒiv aq. destil., placed on the conjunctiva some 15 minutes before the time of operating. The instrument employed was the plain sewing needle No. 10, held in a porte-aiguille, and introduced about an eighth of an inch from the sclerotic margin, into the cornea, and passed to its destination as before directed. The lens was effectually broken up and partly discharged into the aqueous chambers.

October 20th. The solution of the lens is reported as progressing favorably. The needle may require another introduction after several weeks have elapsed.

*October 9th.* CASE VIII.—*Obstruction of the Nasal Duct with Chronic Inflammation of the Lachrymal Sac of the right side.*—Mary W——, married, aged about 35, has been troubled with this affection for now nearly six years. It commenced with weakness of the eye, which was constantly watering; not from a morbid secretion of tears, but from their inability to pass into the nose which was dry; a small tumor then appeared at the inner canthus, and a slight, muco-purulent discharge, which she was first enabled to pass down into the nostril by pressure on the distended lachrymal sac constituting the tumor, now made its appearance in the eye, by regurgitation through the canaliculi. Intercurrent inflammations of the nose and eye were also frequently set up, and the cheek became excoriated by the irritation of the stillicidium lachrymarum.

The sac had been once opened for her relief, but it had closed again, the obstruction returning, to have which removed she has now applied to us.

This condition of things, then, most probably, in the first instance arose from chronic inflammation of the mucous membrane of the lachrymal duct, which resulted in its thickening from the effusion of lymph, between it and its bony walls. Now since the expansion of these is impracticable, the new matter deposited only served to approximate more nearly the internal surface of the membranous parietes of the duct, until, eventually, total occlusion of the passage followed. The natural flow of the tears being thus gradually prevented, acute inflammation of the sac resulted from the stagnation of its distending saline liquors, increased secretion of mucus, and then a pus formation eventuated, and this regurgitating muco-purulence is kept up by subsequent chronic inflammation.

The intercurrent inflammation of the nose resulted from its unaccustomed dryness, or by continuous sympathy—that of the eye from the irritant pus or also by continuous sympathy,—and the excoriation of the cheek was caused by the constant stillicidium lachrymarum as before stated.

The same general cause of symptoms doubtless resulted, upon the closure of the sac after the first operation for its relief.

There is now a daily danger, from the condition of the sac, of a return of acute inflammation, when fistula may result by ulceration from internal pressure on the walls of the distended and occluded cavity. To obviate this, we shall endeavor to re-establish the communication between the eye and nose by the proper operation. This was then performed in the ordinary way. The patient being seated, the tendo-oculi was made tense, the point of the bistoury passed into the sac, and being then directed downwards, outwards, and a little backwards, was made to enter the nose through the duct; a probe was then introduced just before, and whilst the bistoury was withdrawn to render its clearance certain. A wax bougie was passed into the duct immediately upon the withdrawal of the probe; this is to be allowed to remain for several days until the irritation of the parts has subsided, when the style will be substituted. An adhesive strap retains the bougie in its proper position.

On October 16th the case was brought before the class for the second time, and as the irritation resulting upon the operation had totally subsided, the silver nail-headed style was introduced; by it the tears will be enabled at first to reach the nostril, by the capillary attraction exerted in the linear space between the style and the duct walls, and afterwards by the larger space produced by the absorption of the effused lymph, by the pressure upon the membrane thus exerted after the manner of the treatment of any stricture.

A cure may result in from three to six or nine months according as the case may be amenable to treatment. The style must be removed occasionally, cleansed and returned.

A case of *Large Tumor of the superior maxillary bone* of the right side, in a female was then exhibited and some history of it given. The patient will be operated upon on 23d inst.

*October* 13*th.* Cases IX. X. and XI.—Two cases of *amputation of parts of the hand*, from injuries received by circular saws, were exhibited, their histories related, and the effect of the proper treatment, with the progress of the cases, brought to notice, and this great principle of action in these cases enforced, viz: *the necessity of saving all that we can.* Cold water dressings were first employed, followed by cerate and the occasional use of argent. nit., to repress fungous granulations.

Contrasting with these, a case of *amputation of all the toes of both feet*,—which was performed in consequence of the sloughing of the majority of these organs from frost-bite, where the already impaired capillary vessels were further impaired by the supervention of typhus fever,—was next exhibited. Here it was distinctly shown that where the *majority* of the *toes* are to be removed from whatever cause, *all should be taken away*, in order that the base of the column of support may be firm and unwavering. The same treatment was here pursued as in the former cases. The anæsthetic mixture of ether 2 parts, chloroform 1 part, was employed in each of the three cases.

Case XII. In a female aged about 50, a tumor of the size of a hazel-nut, most probably of a commencing malignant character, situated on the inside of the left cheek, was removed with a tenaculum and scapel.

Case XIII.—A small *fibrous sub-integumental tumor*, just below the inner canthus of the left eye, (in an adult, Patrick M'G——,) of about the same size as the preceding, was removed with the scalpel by two elliptical incisions—the edges of the wound being approximated by two fine gold needles and the twisted suture. Some ectropion was produced by this proceeding, which it was supposed would be obviated by the stretching of the extensible integument.

16*th*. On presenting the case to-day, as the lips of the wound were found firmly adherent, the sutures were removed. The slightest possible shade only of eversion was perceptible—the lid will doubtless resume its proper position without delay. On the 20th, perfect union had ensued, and the lid was still improving.

Case XIV.—*An encysted tumor* was removed from the back of the neck of an adult male aged about 40, by a longitudinal incision, two inches in length, immediately over it. The contents of the cyst, of a cheesy character, were discharged, the sac dissected out, and the wound closed by several adhesive strips.

*October* 16*th*. Case XV.—*Enlarged tonsils* were removed. from a child aged five years. Prof. Gilbert has performed the necessary operation on a child aged only three and a half years.

Case XVI.— *The mid-finger of the right hand was removed* with the phalangeal extremity of the metacarpal bone as a remedy for obstinate flexion, rigidity and partial dislocation, from contraction and adhesion of the concerned tendons, the result of abscess. It was done at the request of the patient, a woman engaged in a laborious pursuit, who was incapacitated for her business by its persistence.

On the 20th it was dressed before the class, but the adhesive strips with which the wound was closed were untouched. There had been a little oozing of blood and the nervous symptoms resulting from the operation had demanded a decided anodyne.

Cases XVII. XVIII. XIX.—*Three cases of morbus coxarius*, each presenting one of the three different stages of this affection, were now presented, with appropriate remarks on the disease and its mode of treatment. The paramount indication in these cases was stated to be *quiescence of the joint.* For this purpose Dr. Physick recommended a carved splint. In these cases, in the first and second stages, splints made of binder's board, softened by moistening, and then accurately adapted, from the eighth rib to the knee of the affected side, were provided. These must be worn for a period of nine months at least, probably in the worst case for twelve or fifteen months. For the emaciation in the case in its second stage, the cod liver oil in desert spoonful doses was prescribed.

*October 20th.* Case XX.—*Tumor of the metacarpal bone* of the little finger, was presented as occurring in a boy of twelve years a pupil of Girard College, following upon fracture of the bone, angular union and a consequent exostosis on the palmar aspect.

Case XXI.—*False anchylosis of the elbow* of a child, aged five years, Francis F——, resulting upon a scrofulous abscess about the joint, was exhibited, and the treatment detailed, both general and local.

Case XXII.—A case of *Hernia Infantilis*, non-congenital, but occurring at the sixth month, in a child aged two years, was shown, the nature of the disease explained, and a truss applied.

Previous cases were also shown and dressed—as was also some on every preceding Clinical day.

## Figures and Tables

**Figure f1:**
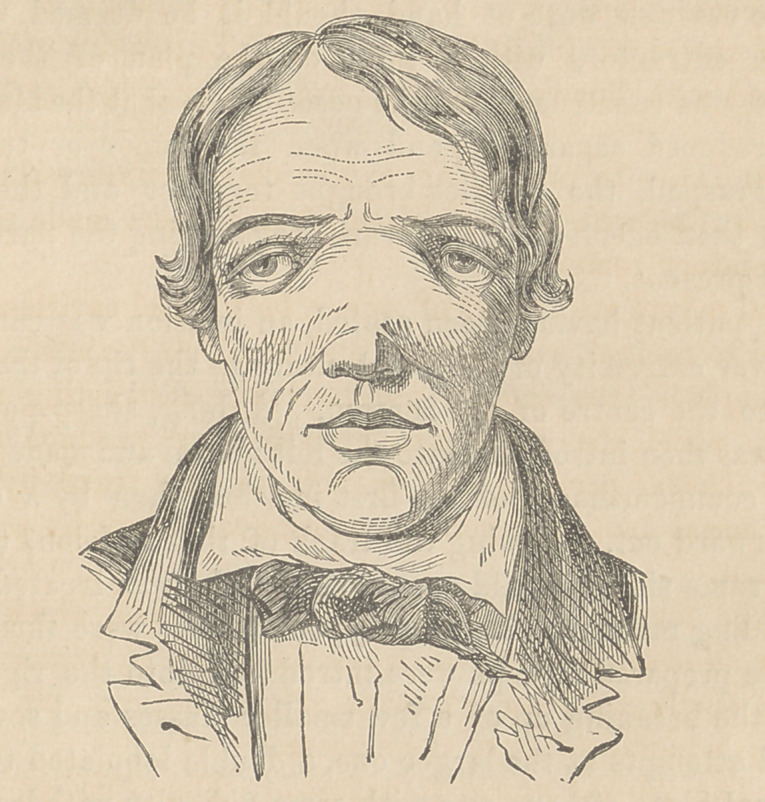


**Figure f2:**